# Characterization of the m6A-Associated Tumor Immune Microenvironment in Prostate Cancer to Aid Immunotherapy

**DOI:** 10.3389/fimmu.2021.735170

**Published:** 2021-08-31

**Authors:** Zezhen Liu, Jiehui Zhong, Jie Zeng, Xiaolu Duan, Jianming Lu, Xinyuan Sun, Qinwei Liu, Yingke Liang, Zhuoyuan Lin, Weide Zhong, Wenzheng Wu, Chao Cai, Guohua Zeng

**Affiliations:** ^1^Department of Urology, Minimally Invasive Surgery Center, Guangdong Key Laboratory of Urology, Guangzhou Urology Research Institute, The First Affiliated Hospital of Guangzhou Medical University, Guangzhou Medical University, Guangzhou, China; ^2^Department of Urology, Guangdong Key Laboratory of Clinical Molecular Medicine and Diagnostics, Guangzhou First People’s Hospital, School of Medicine, South China University of Technology, Guangzhou, China; ^3^Department of Urology, The Second Affiliated Hospital of Guangzhou Medical University, Guangzhou Medical University, Guangzhou, China

**Keywords:** m6A, Tumor Immune Microenvironment, Immunotherapy, PCa, prognosis

## Abstract

The aim of this study was to elucidate the correlation between m6A modification and the tumor immune microenvironment (TIME) in prostate cancer (PCa) and to identify the m6A regulation patterns suitable for immune checkpoint inhibitors (ICIs) therapy. We evaluated the m6A regulation patterns of PCa based on 24 m6A regulators and correlated these modification patterns with TIME characteristics. Three distinct m6A regulation patterns were determined in PCa. The m6A regulators cluster with the best prognosis had significantly increased METTL14 and ZC3H13 expression and was characterized by low mutation rate, tumor heterogeneity, and neoantigens. The m6A regulators cluster with a poor prognosis had markedly high KIAA1429 and HNRNPA2B1 expression and was characterized by high intratumor heterogeneity and Th2 cell infiltration, while low Th17 cell infiltration and Macrophages M1/M2. The m6Ascore was constructed to quantify the m6A modification pattern of individual PCa patients based on m6A-associated genes. We found that the low-m6Ascore group with poor prognosis had a higher immunotherapeutic response rate than the high-m6Ascore group. The low-m6Ascore group was more likely to benefit from ICIs therapy. This study was determined that immunotherapy is more effective in low-m6Ascore PCa patients with poor prognosis.

## Introduction

Prostate cancer (PCa) is a serious threat to men around the world with mortality that is second only to lung cancer (1, 2). Radical surgery and radiation therapy are effective treatment methods for early PCa. Many people lack awareness of the need for PCa screening. Most patients are in advanced or late metastasis stages at the time of diagnosis. As a result, endocrine therapy has become the preferred treatment. However, PCa is likely to enter the drug-resistant stage from the hormone-sensitive stage after approximately 18 months of endocrine therapy. There is still no effective treatment for PCa. Immunotherapy provides a new paradigm in cancer treatment. Marked advances have been made in the field of tumor treatment by immune checkpoint inhibitors (ICIs). However, recent phase II clinical trials (NCT02601014, NCT02787005) revealed that ICIs are only effective for certain patients, and the disease control rate does not exceed 20% in PCa (3, 4). Therefore, improving the defects of ICIs to increase their clinical efficacy is an urgent problem to be solved.

To date, 172 kinds of RNA modifications have been identified. The most common chemical modifications involve N6-methyladenosine (m6A), N1-methyladenosine (m1A) and 5-methylcytosine (m5C). m6A is one of the most abundant modifications in most eukaryotic mRNAs (5, 6). The m6A modification is the methylation of the sixth position of the nitrogen atom of adenosine, with the cellular methyltransferase substrate S−adenosylmethionine serving as the methyl donor for m6A formation (7). The most prevalent RNA methylation, m6A, is a reversible RNA posttranscriptional modification (8, 9). A previous study revealed that m6A modification only occurs on mRNA; however, with the development of detection technology, m6A methylation has been widely found in other types of RNAs such as transport RNA (tRNA) and ribosomal RNA (rRNA) (10). Similar to the modification of DNA, m6A modification is a kind of dynamic reversible process that is regulated by methyltransferases, demethylases and binding proteins, also known as “writers”, “erasers” and “readers”. Methyltransferases, also known as writers, promote m6A methylation modification in RNA (11). The m6A writers include CBLL1, KIAA1429 (VIRMA), METTL14, METTL3, RBM15, RBM15B, WTAP, and ZC3H13. Demethylases (also known as erasers), including FTO and ALKBH5, remove m6A methyl groups from RNA. Binding proteins (also known as readers), including YTHDF1, YTHDF2, YTHDF3, YTHDC1, YTHDC2, RBMX, LRPPRC, IGF2BP3, IGF2BP2, IGF2BP1, HNRNPC, HNRNPA2B1, FMR1, and ELAVL1, can bind to the M6A methylation site in RNA and then perform a specific biological function (12). Accumulating evidence shows that m6A RNA methylation has an outsize effect on RNA production/metabolism and participates in the pathogenesis of multiple diseases, including cancers (5). In a variety of tumors, there is a clear correlation between m6A regulatory factors and patient prognostication, among which the results of some tumor studies show that m6A regulatory factors are related to tumor treatment (13). However, its mechanism of action has yet to be further studied.

A large number of studies have found and confirmed that m6A regulators play an important role in regulating the immune microenvironment of tumors. Fat mass and obesity-associated protein (FTO) plays a role in immune evasion. Ruisu et al. reported two potent small-molecule FTO inhibitors that exhibit strong antitumor effects in multiple types of cancers (14). FTO has a similar antitumor effect on melanoma as a factor in anti-PD-1 resistance (13). The researchers proposed that the combination therapy of FTO inhibitors and anti-PD-1 blockade is beneficial to attenuate resistance to immunotherapy in melanoma patients. More attention has been recently paid to the function of m6A modification in the regulation of circRNAs. Researchers have revealed that YTHDF2, as an m6A reader, sequesters m6A-circRNA and plays an important role in suppressing innate immunity (15). m6A modification, as a reversible epigenetic modification, should be considered in the field of tumor therapy (16). Dali Han et al. showed that YTHDF1 plays an antitumor role through mRNA m6A methylation in dendritic cells (DCs). Studies have shown that the antigen-specific CD8+ T cell antitumor response in YTHDF1-deficient mice is significantly enhanced. The therapeutic efficacy of PD-L1 checkpoint blockade has been markedly improved (17). In melanoma and colorectal cancer, ALKBH5 produces high levels of lactic acid in the tumor microenvironment (TME) and promotes the infiltration of tumor-infiltrating Tregs and myeloid-derived suppressor cells. Inhibiting ALKBH5 could be conducive to antitumor immunotherapy (18). The malignancy, prognosis and antitumor immune response in breast cancer are markedly correlated with the expression pattern of m6A regulators (19).

In the field of PCa, research on m6A regulators is just beginning. Among them, the functions of FTO, YTHDF2, and METTL3 have received special attention. Research on m6A will further reveal the mechanisms of PCa occurrence, progression, and drug resistance. Unlike other cancer types, PCa is a slowly progressing malignancy. The TME plays a substantial role in influencing tumor progression (20). The purpose of this study was to elucidate the relationships among 24 m6A regulatory factors, the immune microenvironment and immunotherapy in PCa and provide a research foundation for the influence of m6A-related epigenetics on the immune microenvironment of PCa.

## Results

### Landscape of 24 m6A Regulators in PCa

**Figure 1:**
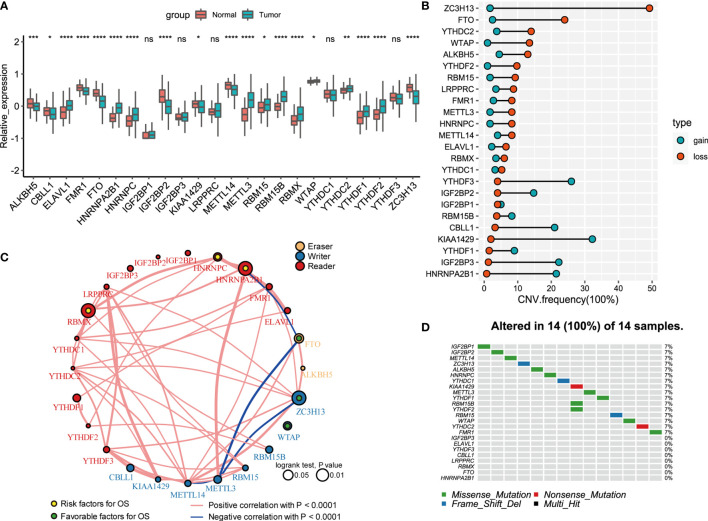
Landscape of genetic and expression variation of 24 m6A regulators in prostate cancer. **(A)** The expression of 24 m6A regulators between normal tissues and prostate cancer tissues. Tumor, red; Normal, blue. The upper and lower ends of the boxes represented interquartile range of values. The lines in the boxes represented median value, and black dots showed outliers. The asterisks represented the statistical p value (*P < 0.05; **P < 0.01; ***P < 0.001; ****P < 0.0001); ns, Not Statistically significant. **(B)** The CNV variation frequency of m6A regulators in TCGA database. The width of the Dumbbell Chart represented the alteration frequency. The deletion frequency, blue dot; The amplification frequency, red dot. **(C)** The interaction between m6A regulators in gastric cancer. The circle size represented the effect of each regulator on the prognosis, and the range of values calculated by Log-rank test was p < 0.01, p < 0.05, respectively. Yellow dots in the circle, risk factors of prognosis; Green dots in the circle, protective factors of prognosis. The lines linking regulators showed their interactions, and thickness showed the correlation strength between regulators. Negative correlation was marked with blue and positive correlation with red. The regulator type Eraser, Writer, Reader was marked with yellow, blue, red respectively. **(D)** The waterfall plot of tumor somatic mutation established for 24 m6a regulators. Each column represented individual patients. The number on the right indicated the mutation frequency in each gene. The down bar plot showed the proportion of each variant type.

A total of 24 m6A regulators, including 8 writers, 2 erasers, and 14 readers, were finally identified in this study. The differential mRNA expression of m6A regulators between PCa tissues and normal tissues was analyzed (**Figure 1A**). Compared to that in normal prostate tissues, the expression level of each eraser (FTO, ALKBH5) was significantly decreased in PCa tissues. Among all the writers, 4 regulators (ZC3H13, METTL14, KIAA1429, CELL1) demonstrated markedly low expression, and 3 regulators (METTL3, RBM15B, RBM15) showed overtly high expression in PCa tissues. For the readers, 7 regulators (ELAVL1, HNRNPA2B1, HNRNPC, RBMX, YTHDC2, YTHDF1, YTHDF2) were expressed at higher levels, and 2 regulators (FMR1, IGF2BP2) were expressed at lower levels in PCa tissues. Then, we summarized the CNVs of the 24 m6A regulators in PCa. Similar to the mRNA expression, the CNV deletions of erasers are shown in **Figure 1B**. Among all writers, 5 regulators (ZC3H13, WTAP, RBM15, METTL3, METTL14) demonstrated CNV deletions, while 3 regulators (RBM15B, KIAA1429, CELL1) showed CNV amplifications. For the readers, 8 regulators (YTHDC2, YTHDF2, LRPPRC, FMR1, HNRNPC, ELAVL1, RBMX, YTHDC1) showed CNV deletions, and 6 regulators (HNRNPA2B1, IGF2BP1, IGF2BP2, IGF2BP3, YTHDF1, YTHDF3) showed CNV amplifications. The analysis of the expression correlation and prognostic significance of the 24 m6A regulators in PCa patients is depicted with the m6A regulatory network shown in **Figure 1C**. We adopted a univariate Cox regression model to calculate the hazard ratios (HRs) and PFS for the m6A regulators (**Table S1**). As a result, RBMX, HNRNPC, and HNRNPA2B1 were considered risk factors, while FTO, ZC3H13, and WTAP were considered favorable factors. Moreover, significant correlations among the expression levels of m6A regulatory factors were detected. The correlation coefficient is positive among RBMX, HNRNPC, and HNRNPA2B1. In contrast, the correlation coefficient between HNRNPA2B1 and FTO is negative. As described above, the trends of methylation or demethylation in PCa are consistent. Furthermore, the frequency of mutations of m6A regulators was near the bottom (**Figure 1D**), which showed no link with tumor development.

The comprehensive analysis of RNA expression, CNV, and PFS can be found in **Table S2**, which pointed to an essential role for FTO, ALKBH5, ZC3H13, HNRNPA2B1, HNRNPC, and RBMX in the prognosis of PCa. Additionally, low expression of erasers, as favorable factors in prognosis, demonstrated that a decrease in m6A demethylation can promote the development of PCa. The upregulation of three readers (HNRNPA2B1, HNRNPC, and RBMX) is associated with poor prognosis in PCa.

### The Characteristics of m6A Regulation Patterns

**Figure 2:**
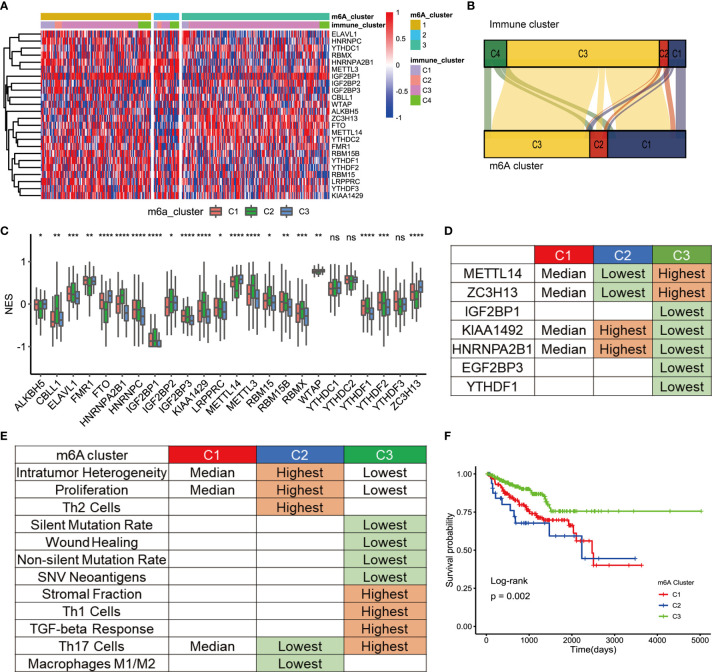
TME cell infiltration characteristic of m6A regulation patterns. **(A)** The cluster was used to identify three expression patterns of 24 m6A regulators in 498 prostate cancers. Standardize the expression of each gene. **(B)** Alluvial diagram showing the changes of m6A regulators clusters and immune clusters of the prostate cancer samples. **(C)** The expression of 24 m6A regulators in three clusters. cluster1, red. cluster2, green, cluster3, blue. The upper and lower ends of the boxes represented interquartile range of values. The lines in the boxes represented median value, and black dots showed outliers. The asterisks represented the statistical p value (*P<005; **P<001; ***P<0001; ****P<0.0001); ns, Not Statistically significant. **(D)** Expression characteristics of 7 key m6a regulators in 3 types of samples. “Highest” means that the mRNA expression of gene is the highest among the three group (p<0.05). “Lowest” means that the mRNA expression of gene is the Lowest among the three group (p<0.05). “Median” means: Compared with the other 2 groups, median is higher than one group (p<0.05) and lower than the other group (p<0.05). **(E)** Key characteristics of three m6a expression clusters. “Highest” means that the mRNA expression of gene is the highest among the three group (p<0.05). “Lowest” means that the mRNA expression of gene is the Lowest among the three group (p<0.05). “Median” means: Compared with the other 2 groups, median is higher than one group (p<0.05) and lower than the other group (p<0.05). **(F)** Survival analyses for the three m6A expression patterns based on 405 patients with prostate cancer from TCGA database including 157 cases in m6A regulators cluster-1, 36 cases in m6A regulators cluster-2, 212 cases in m6A regulators cluster-3. Kaplan-Meier curves with Log-rank p value 0.002 showed a significant survival difference among three m6A expression patterns. The m6Acluster-3 showed significantly better FPI than the other two m6Acluster.

Three distinct modification patterns were eventually identified using unsupervised clustering, including 157 patients in cluster 1, 36 patients in cluster 2, and 212 patients in cluster 3 (**Figure 2A**). To explore the relationship between m6A regulation mode and tumor immunity, we compared 4 kinds of immune patterns and 3 kinds of m6A regulation patterns among 405 PCa patients. Comparative analysis of the relationship between 4 kinds of immune patterns and 3 kinds of m6A regulation patterns is visualized in **Figure 2B**. The results of the chi-square test suggested that there were significant differences among different immune types in the 3 kinds of m6A regulation patterns (chi-square test, p<0.0001, **Figure S2**). Immune subtype C3—characterized by elevated Th17, low to moderate tumor cell proliferation, and lower levels of overall CNVs and aneuploidy than the other immune subtypes—was enriched in most PCa patients. Strikingly, the three distinct methylation modification patterns had distinct proportions of the C3 immune subtype, with m6A regulators cluster 3 having the highest proportion (87.74%), followed by m6A regulators cluster C1 (69.43%) and m6A regulators cluster C2 (33.33%). To determine the key gene markers of m6A regulators clusters, we described the expression of 24 m6A regulators among three clusters (**Figure 2C**). Then, logistic regression analysis was used to determine the markers. Seven key gene markers were jointly involved in the three m6A regulation patterns, including METTL14, ZC3H13, IGF2BP1, KIAA1429, HNRNPA2B1, IGF2BP3, and YTHDF1 (**Table S3**). Moreover, the expression of METTL14 or ZC3H13 was the highest in m6A regulators cluster 3, the lowest in m6A regulators cluster 1 and the middle in m6A regulators cluster 2; there were significant differences among the three groups. In contrast, the expression of KIAA1429 or HNRNPA2B1 was the lowest in m6A regulators cluster 3 and the highest in m6A regulators cluster 2. In addition, the expression of IGF2BP1, IGF2BP3, or YTHDF1 was the lowest in m6A regulators cluster 3 (**Figure 2D**). To determine the immune characteristics of the m6A regulators clusters, we followed the method of Thorsson et al. to calculate the signature score of each tumor sample.

Then, signature scores were compared among the 3 subgroups (**Figure 2E**). Interestingly, m6A regulators cluster 3 had the lowest scores for non-silent mutation rate, SNV neoantigens, ITH, wound healing, and proliferation, which were related to the immune infiltration. In addition, the infiltration of Th1 cells and Th17 cells contribute to antitumor immunity. The scores of Th1 and Th17 were the highest in m6A regulators cluster 3. The score of Th17 cells was the lowest in m6A regulators cluster 2. Stromal fraction was defined as the total non-tumor cellular component. The score of the stromal fraction signature was the highest in m6A regulators cluster 3. A bar chart was used to show the comparison of the signature among the 3 m6A regulators clusters (**Figures S3A-I**). In conclusion, mutations and neoantigens were the main characteristics of m6A regulators cluster 2, and immune cell infiltration was the main feature of m6A regulators cluster 3. The main characteristics of m6A were consistent with the prognostic of the Pca patients. Survival analysis showed that there was a significant difference in prognosis among the three kinds of m6A regulators clusters (**Figure 2F**). The prognosis of m6A regulators cluster 3 was the best. Although there was no significant difference in prognosis between m6A regulators cluster 1 and m6A regulators cluster 2, the prognosis of m6A regulators cluster 1 was more optimistic. This discrepancy may be caused by the small number of patients in m6A regulators cluster 2.

### The Relationship Between the Key Genes and Immune Functions of the Three m6A Subtypes

**Figure 3:**
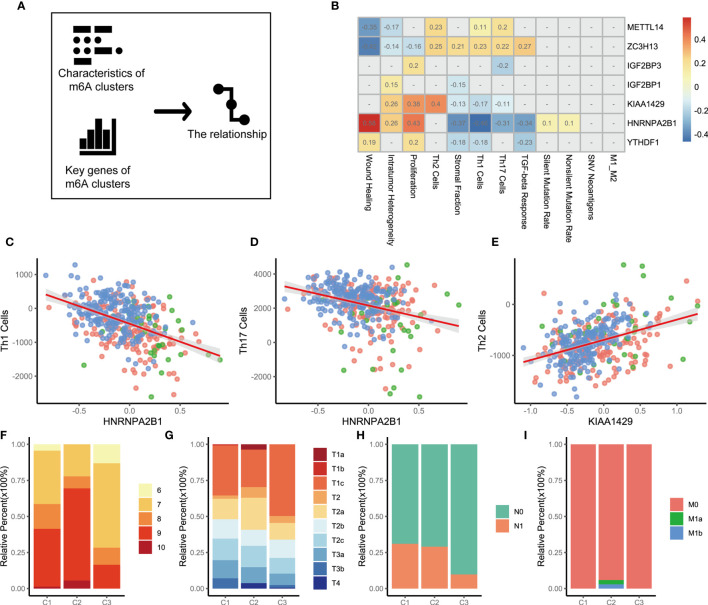
The relationship between m6A genes and characteristics among three m6A regulators clusters. **(A)** The schematic diagram. **(B)** The heatmap shown that correlation between 7 m6A genes and characteristics of m6A regulators clusters. Positive correlation was marked with red. Negative correlation was marked with blue. The correlation coefficient was showed in square box. “NA” means p>0.05. (Spearman test). **(C–E)**. Scatter diagram were showed the correlation between Th1 cells and HNRNPA2B1, Th17 Cells and HNRNPA2B1, Th2 Cells and KIAA1429. **(F)** Differences in Gleason score among three m6A regulators clusters. (p=0.009). **(G)** Differences in distant metastasis among three m6A regulators clusters. (p=0.008) **(H)** Differences in regional lymph node metastasis among three m6A regulators clusters. (p<0.001) **(I)** Differences in primary tumor stage among three m6A regulators clusters. (p=0.015).

The above analysis identified the key genes and immune characteristics of the three m6A regulators clusters. We sought to determine whether these key gene markers were associated with the immune phenotypes of tumors among three m6A regulators clusters (**Figure 3A**). We then examined the specific correlation between 12 immune signature scores and 7 m6A regulators using Spearman’s correlation analyses (**Figure 3B**). The results suggested that key m6A genes were involved in multiple immune-associated signatures, including the TGF-beta response signature, Th1 cell signature, Th17 cell signature, Th2 cell signature and ITH signature. We found that METTL14 and ZC3H13 were positively correlated with Th1 cell signature, Th17 cell signature, and Th2 cell signature. YTHDF1, HNRNPA2B1, KIAA1429, IGF2BP1, and IGF2BP3 showed significant negative correlations. Thus, the effect of METTL14 and ZC3H13 was opposite to that of YTHDF1, HNRNPA2B1, KIAA1429, IGF2BP1, and IGF2BP3 in immunity. Notably, the HNRNPA2B1 and Th1 cell signatures (r=-0.46, p<0.05 **Figure 3C**), HNRNPA2B1 and Th17 cell signatures (r=-0.31, p<0.05 **Figure 3D**), and KIAA1429 and Th2 cell signatures (r=0.40, p<0.05 **Figure 3E**) showed significant correlations. To explore the reasons why m6A regulators cluster 3 has the best prognosis, we utilized GSEA to identify immune-related hallmarks among the m6A regulators clusters. The specific hallmarks are shown in **Figure S4A**. m6A regulators cluster 3 was markedly enriched in full immune activation, such as IL6-JAK-STAT3 signaling and the inflammatory response (**Figure S4B**).

To clarify the potential clinical significance of the m6A regulators clusters, the clinical characteristics of patients in the three m6A regulators clusters were further analyzed. Consistent with the above findings, most patients with high-grade PCa were clustered into m6A regulators cluster 1 and m6A regulators cluster 2 (p=0.009, chi-square, **Figure 3F**). Patients with stage T1 disease accounted for 35.43% of the m6A regulators cluster 2 cohort and 49.70% of the m6A regulators cluster 3 cohort (p=0.015, **Figure 3G**). A similar pattern was observed in regional lymph node metastasis, with an incidence of 29.03% for m6A regulators cluster 2 and 9.77% for m6A regulators cluster 3 (p<0.001, **Figure 3H**). Further analysis revealed that the patients who developed distant metastases were all in the m6A regulators cluster 2 cohort (p=0.008, **Figure 3I**). The above evidence suggests that m6A expression modification is closely related to the development of PCa. We also analyzed cancer-related pathway mutations among the three m6A regulators clusters (m6A regulators cluster 1, **Figure S5A**; m6A regulators cluster 2, **Figure S5B**; m6A regulators cluster 3, **Figure S5C**). A comparison of the mutation frequencies among the three m6A regulators clusters showed that the frequency of mutations in APC, FOXA1, and SPOP was significantly increased in m6A regulators cluster 2 compared with that observed in the other clusters (**Figure S5D**, C1 *versus* C2; **Figure S5E**, C2 *versus* C3). m6A regulators cluster 3, with a better prognosis, showed a dramatically low mutation frequency (**Figure S5E**, C2 *versus* C3; **Figure S5F**, C1 *versus* C3).

The m6A methylation modification patterns and TME immune infiltration were closely related to the prognosis of PCa patients. To further investigate CEGs regulated by m6A methylation modification, we calculated the differential genes among the m6A regulators clusters (p<0.05, |foldchange|>2). The key DEGs were obtained by taking the intersection of the three groups of DEGs. Unsupervised clustering analyses were performed to further validate the m6A modification model based on CEGs. PCa patients were classified into three expression patterns again. We termed these patterns gene cluster 1, gene cluster 2 and gene cluster 3 (**Figure S6A**). PCA confirmed that a significant distinction existed in the m6A transcriptional profiles of the three different gene clusters (**Figure S6B**). As prognostic analysis showed, gene cluster 3 was markedly related to poorer survival, while gene cluster 1 and gene cluster 2 were characterized by prolonged survival (**Figure S6C**). Next, we sought to determine whether there was a relationship between m6A regulators clusters and gene clusters. The results showed that the samples in gene cluster 3 mainly consisted of m6A regulators cluster 1 and m6A regulators cluster 2. In addition, m6A regulators cluster 3 was mainly composed of gene cluster 1 and gene cluster 2 (**Figure S6D**). The expression levels of the 24 m6A regulators among the three m6A regulators clusters are compared in **Figure S6E**. These results indicated that the expression levels of HNRNPA2B1, HNRNPC, and KIAA1429, as markers of the m6A regulators clusters, were remarkably different among the three gene clusters.

**Figure 4:**
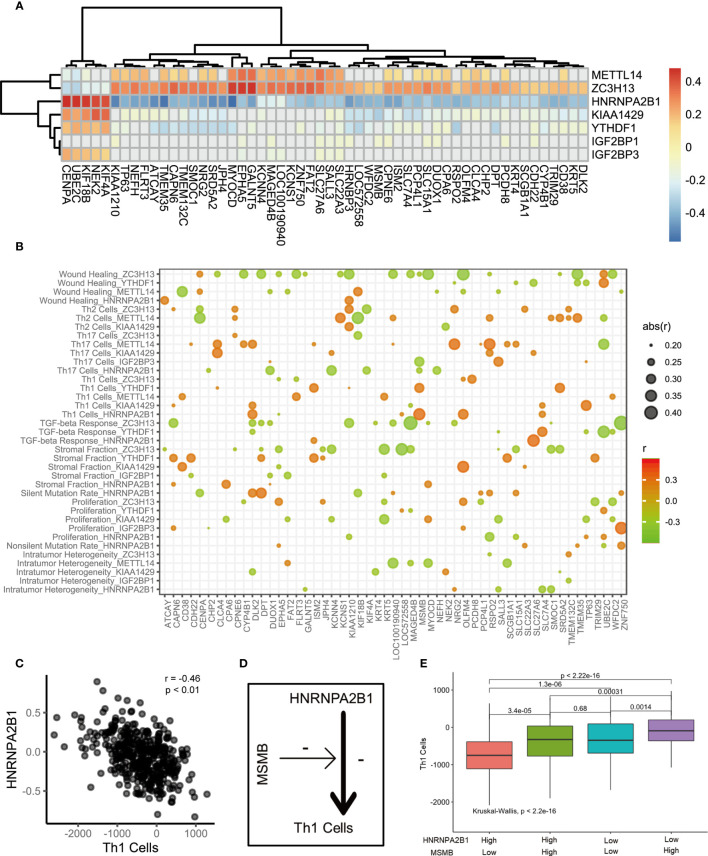
The correlation of m6A regulators, immune cells and m6A associated genes. **(A)** The heatmap represented the correlation between key m6A regulators and key m6A associated genes. positive correlation was marked with red. negative correlation was marked with green. grey means p>0.05. **(B)** Bubble shown mean of correlation coefficient among m6A regulators, immune cells and m6A associated genes. The text on the left indicated the m6A regulators and immune cells. The text on the low represent m6A associated genes. mean of correlation coefficient were marked with size and color of circle. **(C)** The correlation between Th1 Cells and HNRNPA2B1. **(D)** The schematic diagram. **(E)** The median split was used to compare the effects of HNRNPA2B1 and MSMB on Th1 infiltration.

Do m6A regulation patterns lead to immune cell infiltration through CEGs? We first analyzed the relationship between 7 key marker genes and the CEGs (**Figure 4A**). A discernible correlation was observed between METTL14, ZC3H13, HNRNPA2B1, and KIAA1429 and the CEGs. The expression of the CEGs showed a positive correlation with METTL14 and ZC3H13 and a negative correlation with HNRNPA2B1 and KIAA1429. Remarkably, ZC3H13 and HNRNPA2B1 were significantly associated with all related genes. Furthermore, we sought to explore the potential relationships of the CEGs with the immune cell infiltration process through m6A modification. We assume that the existence of CEGs is a necessary condition for biological activities related to the immune functions of m6A regulators. The correlation between m6A regulators and signature scores will be affected by the CEGs. The influence of the CEGs on the biological functions of m6A regulators is depicted with the heatmap shown in **Figure 4B**. HNRNPA2B1 was found to be significantly associated with the progression and prognosis of PCa. Therefore, we used the HNRNPA2B1 and Th1 cell signatures as examples for analysis (**Figure 4C** **)**. First, HNRNPA2B1 is a risk factor for the prognosis of patients with PCa. Decreased HNRNPA2B1 mRNA levels were accompanied by a decrease in Th1 cell scores (r = -0.46, p<0.01, Spearman’s test). MSMB mRNA levels were positively correlated with the HNRNPA2B1 and Th1 cell signature scores, as shown in the heat map. This led to the hypothesis that upregulated expression of MSMB could attenuate the inhibitory effect of HNRNPA2B1 on Th1 cells (**Figure 4D**). The patients were divided into high and low groups according to the median expression levels of HNRNPA2B1 and MSMB. Four groups were classified by pairwise combination. In the high HNRNPA2B1 group, the Th1 cell signature score was elevated when MSMB was overexpressed. Similarly, in the low HNRNPA2B1 group, the Th1 cell signature score was also elevated when MSMB was overexpressed (**Figure 4E**). This result was consistent with the heatmap results. In conclusion, MSMB is a potential factor by which HNRNPA2B1 inhibits Th1 cell infiltration.

### m6A Score Construction

**Figure 5:**
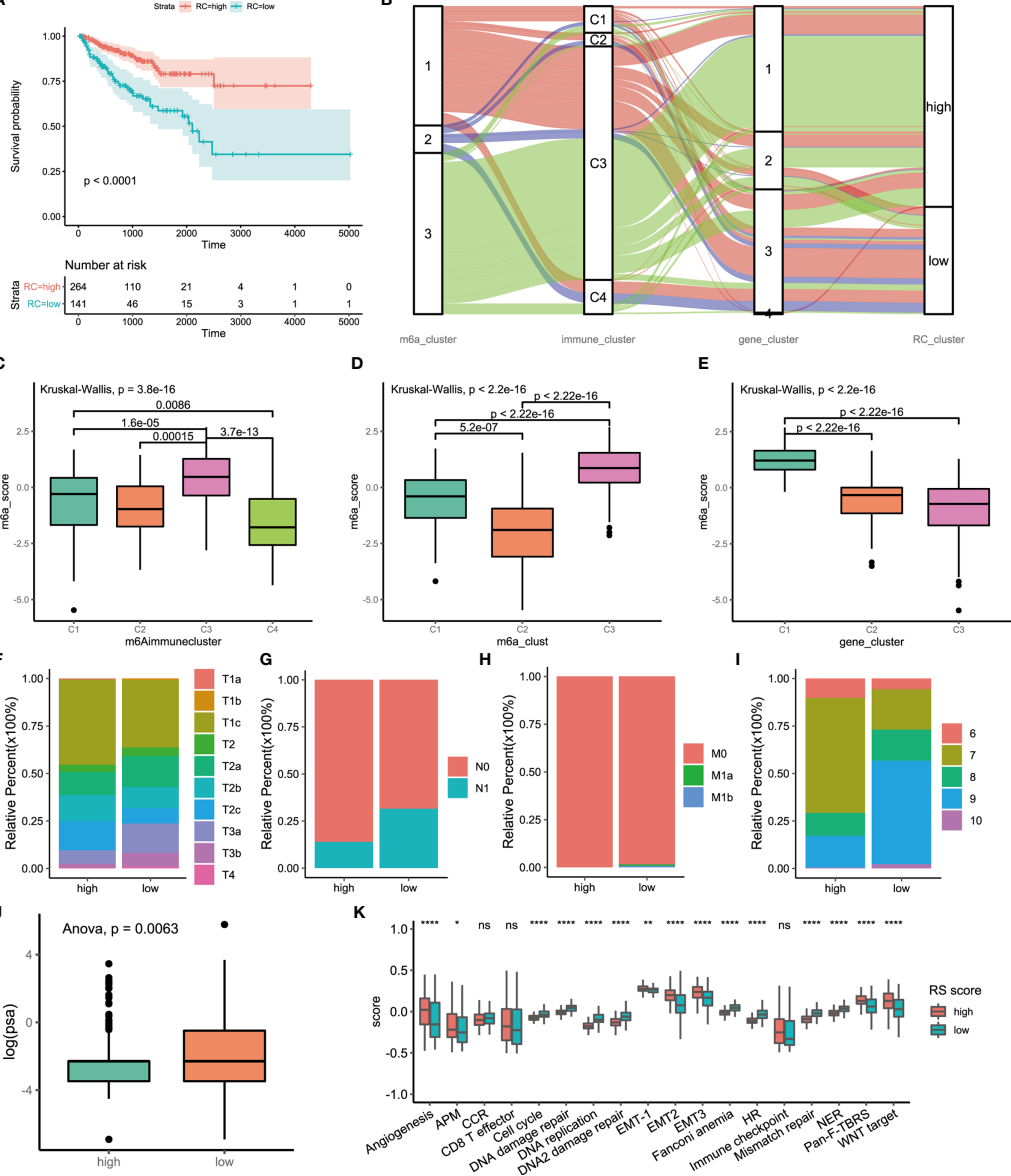
Construction of m6A signatures. **(A)** Survival analyses for low (264 cases) and high (141 cases) m6A risk score patient groups using Kaplan-Meier curves (P < 0.0001, Log-rank test). High risk score group is better than low risk score group. low risk scoring group has a higher risk. **(B)** Alluvial diagram represented the changes of m6A regulators clusters, immune clusters, differential genes clusters and risk score clusters of the prostate cancer samples. **(C)** Differences in m6Ascore among different immune subtypes. **(D)** Differences in m6Ascore among different m6A regulators clusters. **(E)** Differences in m6Ascore among different m6A associated difference genes clusters. **(F)** Differences in primary tumor stage between high and low risk score groups. (p=0.015) **(G)** Differences in regional lymph node metastasis between high and low risk score groups. (p=0.001) **(H)** Differences in distant metastasis between high and low risk score groups. (p=0.1652) **(I)** Differences in Gleason score between high and low risk score groups. (p=0.005) **(J)** Differences in PSA between high and low risk score groups. (p=0.001) **(K)** The score of tumors associated signature between high and low risk scores groups. high risk score group, red. low risk score group, green. The upper and lower ends of the boxes represented interquartile range of values. The lines in the boxes represented median value, and black dots showed outliers. The asterisks represented the statistical p value (*P<005; **P<001; ****P<0.0001); ns, not statistically significant. APM, Antigen processing machinery; CCR, Cell cycle regulators; HR, Homologous recombination; NER, Nucleotide excision repair.

The above analyses were based on m6A methylation modification and could accurately reflect the expression pattern of m6A regulators in PCa. Considering the need for more accurate and clear models for immunotherapy prediction, based on these phenotype-related genes, we constructed a set of scoring systems to quantify the m6A modification pattern of individual patients with PCa, which was termed the m6Ascore. The cluster of m6A regulators and the CEGs were classified into three categories, but two distinct prognoses were demonstrated by survival analysis. We sought to determine whether there was a potential link between gene expression modification and prognostic classification. Therefore, PCA of CEGs was used to distinguish high and low m6Ascore. Then, clinical characteristics and prognosis were investigated. Kaplan-Meier survival analysis indicated that the high-m6Ascore group had a better prognosis than the low-m6Ascore group (**Figure 5A**). We analyzed the relationships among the m6A regulators clusters, immune clusters, gene clusters and m6Ascore groups (**Figure 5B**). A total of 95.83% of patients in m6A regulators cluster 3, with good prognosis, were included in the high-m6Ascore group. A total of 86.11% of patients in m6A regulators cluster 2, with poor prognosis, were included in the low-m6Ascore group. A total of 63.28% of patients in gene cluster 3 had a poor prognosis and belonged to the low-m6Ascore group. The m6Ascore could effectively reflect the expression pattern of m6A regulators and CEGs. We further analyzed the distribution of the m6Ascore in immune clusters, m6A regulators clusters and gene clusters. Immune cluster 3 showed a markedly increased m6Ascore compared to the other clusters (**Figure 5C**). m6A regulators cluster 3 showed the highest m6Ascore, while m6A regulators cluster 2 had the lowest m6Ascore (**Figure 5D**). In addition, gene cluster 1 had the highest m6Ascore (**Figure 5E**). The bubble plot reflects the difference in m6A regulators (**Figure S7A**), CEGs (**Figure S7B**) and immune-associated signatures (**Figure S7C**) between the high- and low-m6Ascore groups.

There were significant differences in the clinical characteristics between the two groups. The proportion of less invasive lesions (T1 and T2 stage) was significantly higher in the high-m6Ascore group (p=0.01499, Fisher’s exact test) (**Figure 5F**). The percentage of lymph node metastasis was lower in the high-m6Ascore group (p=0.00014, Pearson’s chi-squared test) (**Figure 5G**). Distant metastasis of PCa occurred in only the low-m6Ascore group (p=0.1278, Fisher’s exact test) (**Figure 5H**). The Gleason score (GS) is an established predictor of progression risk in treated and untreated PCa patients. Subgroup analysis for the GS of PCa showed that the proportion of low-grade cancer (GS ≤ 7) in the high-m6Ascore group was higher than that in the low-m6Ascore group (p=0.0005, Fisher’s exact test) (**Figure 5I**). The prostate-specific antigen (PSA) concentration in the high-m6Ascore group was lower than that in the low-m6Ascore group (**Figure 5J**). Furthermore, tumor-associated biological functions were compared (**Figure 5K**). In summary, the m6Ascore reflects not only the m6A modification model but also PCa prognosis. Therefore, it is a concise and explicit scoring system.

### The Role of m6A Regulation Patterns in Anti-PD-1/L1 Immunotherapy

**Figure 6:**
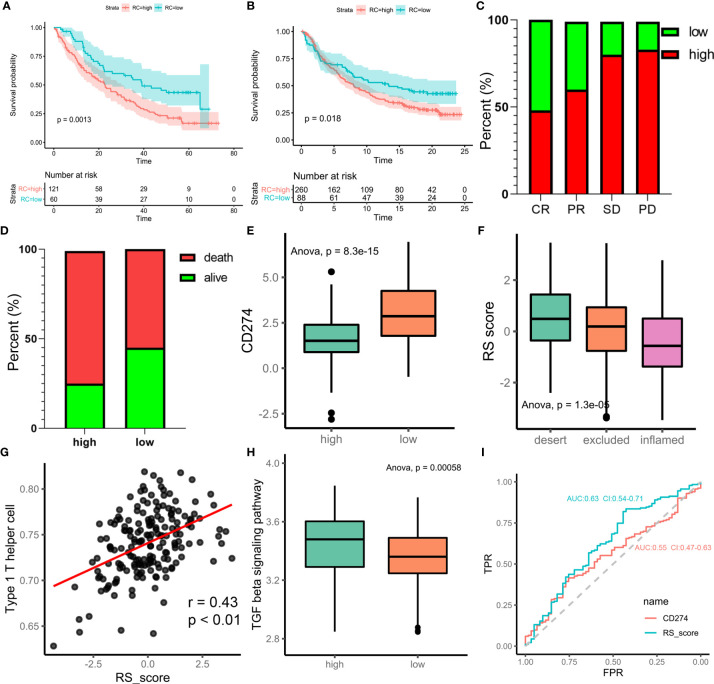
m6A regulation patterns in the potential role of anti-PD-1/L1 immunotherapy. **(A)** Survival analyses for low-risk scores (60 cases) and high-risk scores (121 cases) using Kaplan-Meier curves (patients with Advanced ccRCC enrolled in prospective clinical trials of treatment with PD-1 blockade. P = 0.0013, Log-rank test). The prognosis of immunotherapy in high-risk scores cohort is poor. **(B)** Survival analyses for low-risk scores (88 cases) and high-risk scores (260 cases) using Kaplan-Meier curves (IMvigor210 cohort. P = 0.018, Log-rank test). **(C)** Differences of m6Ascore among CR, PR, SD, PD. (CR, complete response. SD, stable disease. PD, progressive disease. PR, partial response.) **(D)** Proportion of deaths difference between high and low risk scores. (Chi-square test, p<0.0001) **(E)** Differences of CD274 between high and low risk score groups. **(F)** Difference of risk score among tumor immune micro-environments (desert, excluded and inflamed). **(G)** scatter diagram shown the correlation between Type 1 T helper cell and risk score. **(H)** Difference score of TGF beta signaling pathway between high and low of m6Ascore in TCGA prostate cancer database (high m6Ascore group marked with green. low m6Ascore group marked with red. t.test, p<0.001). **(I)** Receiver operator characteristic curve (ROC) of CD274 and RS score represented prediction efficiency of anti-PD-1/PDL1 immunotherapy by risk score.

Immunotherapy is a major breakthrough in cancer therapy. Based on the relationship between m6A modification and immune cell infiltration in PCa, we further investigated whether m6A modification is related to the effect of tumor immunotherapy. Two immunotherapy datasets were used for analysis (IMvigor210 and GSE78220). Interestingly, similar results were obtained from the two datasets, in which patients treated with ICIs in the low-m6Ascore group showed better survival outcomes (IMvigor210, **Figure 6A**; GSE78220, **Figure 6B**). This finding is contrary to previous results showing that patients with a high m6A score have a better prognosis. Taking IMvigor210 as an example, we further analyzed the response to immunotherapy. The response rate of immunotherapy in the low-m6Ascore group was significantly higher than that in the high-m6Ascore group. The proportions of patients with a high m6Ascore who achieved complete remission (CR), partial remission (PR), stable disease (SD), and progressive disease (PD) were 48%, 60%, 80%, and 83%, respectively (p < 0.0001, chi-square test) (**Figure 6C**). The prognosis of immunotherapy showed a marked difference between the high- and low-m6Ascore groups (p<0.0085, chi-square test) (**Figure 6D**). The significant therapeutic advantages and clinical response to anti-PD-1/L1 immunotherapy in patients with a low m6Ascore compared to those with a high m6Ascore were confirmed. Moreover, patients with high m6Ascore showed significantly high CD274 expression, which indicated a potential effective response to anti-PD-1/L1 immunotherapy (**Figure 6E**). We investigated the difference in m6Ascore among the three immune phenotypes and found that the desert phenotype had a higher m6Ascore, and immune checkpoint inhibitors had difficulty exerting antitumor effects on this phenotype (p<0.0001, ANOVA) (**Figure 6F**). Type 1 T helper cells, which are essential for regulating the immune microenvironment of PCa, were positively correlated with the m6Ascore (r=0.43, p<0.01) (**Figure 6G**). In PCa, activation of the TGF-beta signaling pathway is an important factor that prevents the antitumor effects of ICIs. We compared the score of the TGF-beta signaling pathway between the high- and low-m6Ascore groups in the immunotherapy datasets. The results were in line with the theoretical expectations. The TGF-beta signature score was lower in the low-m6Ascore group, where immunotherapy was more effective (**Figure 6H**). The ROC curve revealed that the m6Ascore could be a potential biomarker for prognosis and the clinical response assessment of immunotherapy (AUC of m6ascore:0.63(0.54-0.71), AUC of CD274 = 0.55(0.47-0.63). DeLong’s test. p value=0.9287) (**Figure 6I**). The m6ascore has the same predictive power as CD274.

## Discussion

Currently, increasing attention has been given to m6A modification and tumor progression. Growing evidence suggests that m6A regulators have important functions in regulating the TME in gastric cancer (21), pancreatic adenocarcinoma (22, 23), and breast cancer (19). This study was designed to investigate the potential functions of m6A modification in the immune microenvironment of PCa and identify the m6A regulation patterns that would benefit from ICI therapy. Our findings will contribute to individualized immunotherapy for patients with PCa.

m6A regulators are highly involved in cancer progression, including tumorigenesis (24), angiogenesis (25), and metastasis (26). Accumulating evidence indicates that m6A modification often plays a dual role (27, 28). On the one hand, aberrant m6A methylation may result in both the upregulation of oncogenes and the silencing of tumor suppressor genes, which fosters cancer progression. On the other hand, m6A RNA methylation can be regulated by the expression of m6A regulators and the activity of m6A enzymes, thereby further affecting tumor progression. Based on the interactions among m6A regulators in PCa, we revealed the trends of m6A readers, writers and erasers. The expression levels of all erasers were decreased, while the expression levels of a majority of readers were significantly increased. This trend indicates that the m6A RNA methylation process was enhanced in PCa. Furthermore, these factors regulate immune cell infiltration in PCa.

A previous study revealed three distinct TME cell infiltration characterizations based on m6A methylation modification patterns in gastric cancer (21). Of them, the immune-excluded phenotype was characterized by the activation of innate immunity and stroma; the immune-inflamed phenotype was characterized by the activation of adaptive immunity; and the immune-desert phenotype was characterized by the suppression of immunity. Here, based on the m6A transcriptional profile, unsupervised clustering also revealed three fully distinct patterns of m6A modification in the PCa cohort. Surprisingly, m6A regulation patterns were highly correlated with TME cell infiltration characteristics. It is well demonstrated that METTL14 can form stable complexes with METTL3, and function as a pseudo-methyltransferase that stabilizes METTL3 and recognizes target RNA. Currently, the roles of “writer” genes METTL3 and METTL14 have been explored by various studies. It is reasonably thought that proteins in the m6A methyltransferase complex should exhibit similar functions. This is not always the case as reported. These two “writer” genes, METTL3 and METTL14, showed an opposite pattern of expression in tumors compared to normal. Similar result exists in live cancer. This phenomenon suggests that METTL3 and METTL14 exerted as a complicated regulator in prostate cancer. We will conduct further exploration and research of these matters in follow-up work. Accumulating evidence in recent years reveals that METTL3 plays key roles in a variety of cancer types. METTL3 is upregulated in PCa tissues, especially those with bone metastasis across multiple studies (29–31). While METTL3 exhibits oncogenic functions in most cancer types, it was also reported as a tumor suppressor in RCC, bladder cancer, glioblastoma stem cell. METTL3 was reported to play either oncogenic or/and tumor-suppressive functions by different groups, which may be explained by tumor heterogeneity and/or different model systems used for the study, and further comprehensive and detailed studies are warranted to gain a better view. In addition, ZC3H13 suppresses proliferation and invasion by inactivating Ras-ERK signaling in colorectal cancer (32). Similarly, the expression of METTL14 and ZC3H13 was associated with slow disease progression in PCa. In contrast, KIAA1429 and HNRNPA2B1 were highly expressed in m6A regulators cluster 2, which was linked to worse clinical outcomes. KIAA1429 has been indicated to be an oncogene in liver cancer (33), breast cancer (34), gastric cancer (35), and osteosarcoma (36). HNRNPA2B1 is significantly upregulated in PCa and impacts disease progression. At present, there are few studies on HNRNPA2B1-mediated m6A modification in PCa; however, the effect of HNRNPA2B1 on immune cell infiltration has gradually been noticed.

This study synthetically analyzed the TME immune cell infiltration characterization of distinct m6A methylation modification patterns and the m6A transcriptional profile. m6A regulators cluster 3, with high expression of METTL14 and ZC3H13, was characterized by increased Th1 cells, Th17 cells, stromal fraction and TGF-beta response. Among them, an increase in stromal fraction indicates less malignancy. Antitumor immune responses are triggered by Th1 cells. Th17 cells are a favorable prognostic indicator in PCa (37). Additionally, Th17 cells are related to the efficacy of PD-1 blockade treatment in PCa (38).

Here, the survival analysis results suggested that patients with high Th17 infiltration showed a significantly better prognosis in PCa. We speculated that the infiltration of Th1 cells and Th17 cells contributes to the good clinical outcomes of m6A regulators cluster 3. However, the TGF-beta response that is also upregulated in m6A regulators cluster 3 deserves closer attention. Shiping Jiao et al. found that TGF-beta can inhibit the Th1 response, thus reducing the effect of ICIs (39–41). Surprisingly, this is consistent with our results regarding the efficacy of immunotherapy. m6A regulators cluster 3 was mainly involved in the high m6Ascore group, which was related to better, marked treatment outcomes. This might be a pivotal reason for poor responses to immunotherapy in m6A regulators cluster 3.

Recent findings have shown that m6A regulators play important and diverse biological functions by directly or indirectly regulate expression of downstream. The downstream reflect the biological function of m6A regulators. To better understand the role of m6A regulators in prostate cancer we performed the analysis of downstream. Results reveal that HNRNPA2B1 and ZC3H13 play an essential role. Multiple studies have confirmed that HNRNPA2B1 closed related to prostate cancer progression (42–44). Whereas, the two have opposite effects on downstream. Th1 signaling and survival analysis were consistent with this finding in our study. Further analysis found that MSMB is a key intermediate factor. Currently, the relationship between HNRNPA2B1 and ZC3H13 is unclear. These results suggested that the two may be balanced with each other to act on downstream genes to jointly regulate prostate cancer progression. The detailed mechanisms need further exploring and investigating in the future.

This study conducted cluster analysis of multiple dimensions in PCa, including m6A regulators clusters, immune clusters and CEGs clusters. The expression of 24 m6A regulators was classified into three distinct m6A regulation patterns. The interactions among m6A regulators could carry out the function of m6A methylation or demethylation. The m6A regulators clusters indicated that tumor cells regulate their function in different RNA regulation patterns. The CEGs clusters were assessed to explore potential regulatory factors that exert their biological functions in different m6A expression patterns. In other words, the CEGs clusters reflect the biological functions related to m6A regulator expression patterns. Survival differences in distinct patterns suggest that m6A regulation patterns have a significant effect on the survival of PCa patients. However, the classification of three m6A regulators clusters cannot clearly reflect the prognosis of patients. Therefore, m6Ascore was used to not only distinguish the m6A expression patterns but also directly reflect prognosis.

Our results revealed that the m6Ascore was related to the effect of immunotherapy. Among those who did not receive immunotherapy, the high m6Ascore group had a better prognosis. In comparison, patients who received immunotherapy who were in the low m6Ascore group also had a better survival outcome. The potential cause of this result was the higher mutation rate of the low m6Ascore group, accompanied by the release of SNV neoantigens and ITH. The higher the mutation rate in tumor tissue is, the more accumulated immunogenicity is released, and the better the treatment efficacy (45, 46). As important tumor antigens for the human immune system, neoantigens have emerged from studies of novel ICIs targeting CTLA4 and PD1, which are expressed by activated T cells (47, 48). Bojan Losic et al. found that the area with the lowest tumor purity level is the area with the highest extent of immune infiltration. The degree of immune infiltration correlates well with ITH in liver cancer (49). This is consistent with our observation in PCa. However, they also found an inverse association between ITH and the immune checkpoint response in liver cancer. Conversely, we concluded that patients with high tumor heterogeneity responded better to immunotherapy. A few possible reasons may explain this. On the one hand, tumor heterogeneity responds differently to immunoreactivity (50–52). On the other hand, the immunotherapy response is not solely determined by tumor heterogeneity.

In conclusion, our work demonstrated three m6A regulation patterns for PCa and identified the characteristics of the transcriptome and immune infiltration in individual m6A regulation patterns. This study not only describes the functions of m6A regulators but also reveals the underlying causes of different clinical outcomes and immunotherapy responses in distinct m6A regulation patterns. A comprehensive evaluation of individual m6A regulation patterns will contribute to enhancing our deep understanding of immune-cell characterization of PCa and help us to develop personalized immunotherapy strategies to manage PCa patients.

## Method

### PCa Dataset Source

The workflow of our study is shown in **Figure S1**. The RNA sequencing (RNA-seq) (https://tcga.xenahubs.net/download/TCGA.BRCA.sampleMap/HiSeqV2_PANCAN.gz), gene mutation and clinical data of 495 PCa patients were extracted from The Cancer Genome Atlas (TCGA) dataset (http://cancergenome.nih.gov/). The copy number variation (CNV) data were downloaded from Broad GDAC Firehose (https://gdac.broadinstitute.org/). Immune cell fraction data were downloaded from CIBERSORT (https://cibersort.stanford.edu/).

### Unsupervised Clustering of 24 m6A Regulators

The 24 m6A regulators included 8 writers (WTAP, KIAA1429, CBLL1, RBM15, RBM15B, ZC3H13, METTL3, and METTL14), 2 erasers (FTO and ALKBH5) and 14 readers (ELAVL1, FMR1, HNRNPA2B1, HNRNPC, IGF2BP1, IGF2BP2, IGF2BP3, LRPPRC, RBMX, YTHDC1, YTHDC2, YTHDF1, YTHDF2, and YTHDF3). Model-based clustering analysis was applied to identify m6A regulation patterns based on the expression of 24 m6A regulators for further analysis (model-based clustering: https://www.rdocumentation.org/packages/mclust/versions/5.4.6/topics/Mclust).

### Identification of the Characteristics of Distinct m6A Regulators Clusters

Differences in characteristics between groups were analyzed using the Kruskal–Wallis test with Dunn *post hoc* tests (for continuous variables, R package FSA). We defined an index score as “highest” when it was the highest among all clusters and as “lowest” when it was the lowest. We defined an index score as the “median” when the score of one group was between those of the other two groups, with significant differences. P value less than 0.05 was regarded as a significant difference.

### Gene Set Enrichment Analysis (GSEA) and Biological Function Analysis

To better understand the different biological functions of m6A modifications, GSEA was performed to functionally annotate genes (18). The gene sets of “h.all.v7.2.symbols.gmt” were downloaded from the Molecular Signatures Database (MSigDB) for GSEA (https://www.gsea-msigdb.org/gsea/index.jsp). The GSEA R package was used to compute the enrichment scores and simulated enrichment scores. Adjusted P values less than 0.05 were considered statistically significant.

### Analysis of Somatic Mutations

Somatic mutation data were downloaded from the GDC data portal (https://portal.gdc.cancer.gov/). The “Masked Somatic Mutation” data were selected. The maftools R package provides multiple analysis modules to perform the visualization process (19).

### Estimation of TME Cell Infiltration

CIBERSORT is a deconvolution algorithm for quantifying cell fractions from bulk tissue gene expression profiles that was reported by Newman et al. (20). CIBERSORT can accurately estimate the immune composition of a tumor biopsy. The relative abundances of immune cells and immune-associated signatures were quantified by Thorsson et al. with CIBERSORT. The CIBERSORT results were derived from the website (https://gdc.cancer.gov/about-data/publications/panimmune) (21). The relative abundance of Th1/Th2/Th17 cell infiltration in the PCa microenvironment was calculated by the single-sample gene set enrichment analysis (ssGSEA) algorithm. The relative abundance of TME cell infiltration was used to estimate the relationship between immune cells and progression-free survival (PFS) or overall survival (OS) by univariate Cox analysis and differential expression among m6A subclusters.

### Generation of the m6Ascore

To identify the most suitable evaluation index of m6A regulation patterns in PCa, we constructed multiple evaluation criteria, including m6A regulators clusters, gene clusters and the m6Ascore. The m6A regulators clusters and gene clusters were obtained from unsupervised clustering. The gene clusters were identified to verify the stability of the m6A regulators clusters by the consensus clustering algorithm. The m6Ascore is a set of scoring systems to evaluate the m6A regulation patterns of individual patients with PCa. The procedures for the construction of the m6A gene signature were as follows. The differentially expressed genes (DEGs) of individual m6A regulators clusters were screened using the limma R package. Overlapping genes that were extracted from three different m6A regulators clusters were identified as key DEGs. Principal component analysis (PCA) was used to construct the m6Ascore with the key DEGs. Both principal components 1 and 2 were selected as signature scores. This method makes it easier to evaluate m6A regulation patterns in individual PCa patients. In the formula, “i” represents m6A phenotype-related genes.

$$m6\mathrm{score}=\sum ({\mathrm{PC}1}_{i}+{\mathrm{PC}2}_{i})$$

### Multiple Co-Expression Analysis

The relationship between m6A regulators and the characteristics of the tumor immune microenvironment (TIME) in PCa was confirmed in our study. To further investigate the influence of the co-expression genes (CEGs) of the 24 m6A regulators on the relationship between m6A regulators and the characteristics of the TIME, multiple co-expression analysis was performed.

The m6A modification model-associated m6A regulators and m6A modification model-associated immune signature were obtained from the characteristics of distinct m6A regulators clusters. The m6A regulators included METTL14, ZC3H13, IGF2BP1, KIAA1429, HNRNPA2B1, IGF2BP3, and YTHDF1. The signatures included Th2 cells, silent mutation rate, wound healing, intratumor heterogeneity (ITH), non-silent mutation rate, proliferation, single-nucleotide variant (SNV) neoantigens, stromal fraction, Th1 cells, TGF-beta response, Th17 cells, and macrophage I/macrophage II (m1_m2). The overlapping genes that were extracted from the DEGs of three m6A regulators clusters (54 genes) were regarded as CEGs.

The procedures for multiple co-expression analysis were as follows. Step 1: 60% of the 405 samples were randomly selected for subsequent analysis. Step 2: the correlations between the expression of 7 regulators and signature scores were calculated using Spearman correlation analysis. P values less than 0.05 were considered relevant. The correlation coefficient was recorded as r1. The median expression levels of the m6A-associated genes were calculated for further analysis. Step 3: 100 medians and r1s were obtained by repeating step 1 and step 2 100 times. The correlation coefficients and P values between the 100 medians and r1s were calculated with Spearman correlation.

### Statistical Analysis

For quantitative data, comparisons between two groups were analyzed by t-test, Student’s t-test or the Wilcoxon signed-rank test. The Kruskal-Wallis test and ANOVA were used for the assessment of multiple groups. The chi-square test was performed to analyze qualitative data. Spearman’s correlation was calculated for the correlation of different mRNA and protein expression levels.

The survminer R package was used to determine the cut-off point of each dataset subgroup. The “surv-cutpoint” function, which repeatedly tests all potential cut points to find the maximum rank statistic, was applied to dichotomize the m6Ascore. Then, the patients were divided into high- and low-m6Ascore groups based on the maximally selected log-rank statistics to decrease the batch effect of the calculation. The Kaplan-Meier method and log-rank test were used to generate the survival curves of the prognostic analysis. The specificity and sensitivity of the m6Ascore were assessed through receiver operating characteristic (ROC) curves, and the area under the curve (AUC) was quantified using the pROC R package. All the tests were two sided, and P < 0.05 was regarded as statistically significant. All of the analyses were performed with R software (version 4.01, http://www.R-project.org).

## Data Availability Statement

The original contributions presented in the study are included in the article/**Supplementary Material**. Further inquiries can be directed to the corresponding authors.

## Author Contributions

ZZL and JHZ designed this work. ZZL, JHZ, XD, and YL integrated and analyzed the data. ZZL and JZ wrote this manuscript. ZZL, CC, XD, JL, XS, QL, YL, ZYL, WZ, and GZ edited and revised the manuscript. All authors contributed to the article and approved the submitted version.

## Funding

This study was funded by the China Postdoctoral Science Foundation (No. 2020M672590), the National Natural Science Foundation of China (No. 81870483, 81872437, 82072813 and 82073294), the Science and Technology Planning Project of Guangdong Province (No. 2017B030314108), Scientific research projects in colleges and universities of Guangzhou Education Bureau (No. 201831811), Guangzhou Municipal Science and Technology Project (No. 201803040001) and General Research Projects of Guangzhou Science and Technology Department (No. 201804010053), Medical Research Foundation of Guangdong Province (No. A2019176).

## Conflict of Interest

The authors declare that the research was conducted in the absence of any commercial or financial relationships that could be construed as a potential conflict of interest.

## Publisher’s Note

All claims expressed in this article are solely those of the authors and do not necessarily represent those of their affiliated organizations, or those of the publisher, the editors and the reviewers. Any product that may be evaluated in this article, or claim that may be made by its manufacturer, is not guaranteed or endorsed by the publisher.

## Supplementary Material

Supplementary Figure 1 Flowchart depicting the workflow of this study.

Supplementary Figure 2 Table shown that relationship between m6A regulators clusters and immune clusters in the prostate cancer. colnames are immune clusters. rownames are m6A regulators clusters.

Supplementary Figure 3 The histogram shows the comparison of 12 characteristic scores among three m6A regulators clusters.

Supplementary Figure 4 **(A)** Venn Diagram showed that 9 hallmarks were obtained from the intersection. C1_C2 represent the different hallmark between cluster1 and cluster2. C1_C3 represent the different hallmark between cluster1 and cluster3. C2_C3 represent the different hallmark between cluster2 and cluster3. **(B)** GSEA analysis of 50 hallmarkers.

Supplementary Figure 5 **(A)** Cluster1. The first column represented tumor associate signaling pathway. The second column shown the number and proportion of mutated genes in signaling pathway. The third column indicated mutation patients. **(B)** Cluster2. **(C)** Cluster3. **(D)** The Cluster3 is compared with the Cluster1. Forest plot shown differences of mutation genes among three m6A regulators clusters. median column represented gene mutation frequencies. (** p<0.01; ***p<0.001) **(E)** The Cluster3 is compared with the Cluster2. **(F)** The Cluster3 is compared with the Cluster1.

Supplementary Figure 6 Unsupervised clustering of overlapping m6A expression patterns differential genes. **(A)** Three clusters were identified by unsupervised clustering base on differential genes overlapping of three m6A expression patterns. **(B)** Principal component analysis shown a remarkable difference among three differential genes clusters. **(C)** Survival analyses for the three differential genes clusters based on TCGA database including 165 cases in differential genes cluster-1, 76 cases in differential genes cluster-2, 162 cases in differential genes cluster-3. Kaplan-Meier curves with Log-rank p value <0.0001 showed a significant survival difference among three differential genes clusters. The cluster-3 showed significantly badly FPI than the other two cluster. **(D)** Alluvial diagram showing the changes of m6A regulators clusters and differential genes clusters of the prostate cancer samples. **(E)** The expression of 24 m6A regulators in three clusters. cluster1, red. cluster2, green, cluster3, blue. (*P<005; **P<001; ***P<0001; ****P<0.0001).

Supplementary Figure 7 **(A)** bubble shown difference in key characteristics between high and low risk score groups. -log10(adjust p value) were marked with color. log(foldchange) were marked with size. **(B)** bubble shown difference in expression of 24 m6A genes between high and low risk score groups. **(C)** bubble shown difference in expression of m6A associated genes between high and low risk score groups.

## Abbreviations

CNV, Copy number variation; DC, Dendritic cell; DEGs, Differentially expressed genes; EMT, Epithelial-mesenchymal transition; GDC, Genomic Data Commons; GSEA, Gene set enrichment analysis; ICB, Immunological checkpoint blockade; ITH, Intratumor heterogeneity; m6A, N6-methyladenosine; MSigDB, Molecular Signatures Database; NK cell, Natural killer cell; PFS, Progression-free survival; PCA, Principal component analysis; SNV, Single nucleotide variant; ssGSEA, Single-sample gene-set enrichment analysis; TIME, Tumor immune microenvironment; PSA, Prostate-specific antigen; PCa, Prostate cancer; ICIs, Immune checkpoint inhibitors; TME, Tumor microenvironment; GS, Gleason score; CR, Complete remission; PR, Partial remission; SD, Stable disease; PD, Progressive disease.
