# Supplementary figures and images for: Characterization of the m6A-Associated Tumor Immune Microenvironment in Prostate Cancer to Aid Immunotherapy
Source: Front Immunol. 2021 Aug 31;12:735170. doi: 10.3389/fimmu.2021.735170 (PMC8438522; doi:10.3389/fimmu.2021.735170)

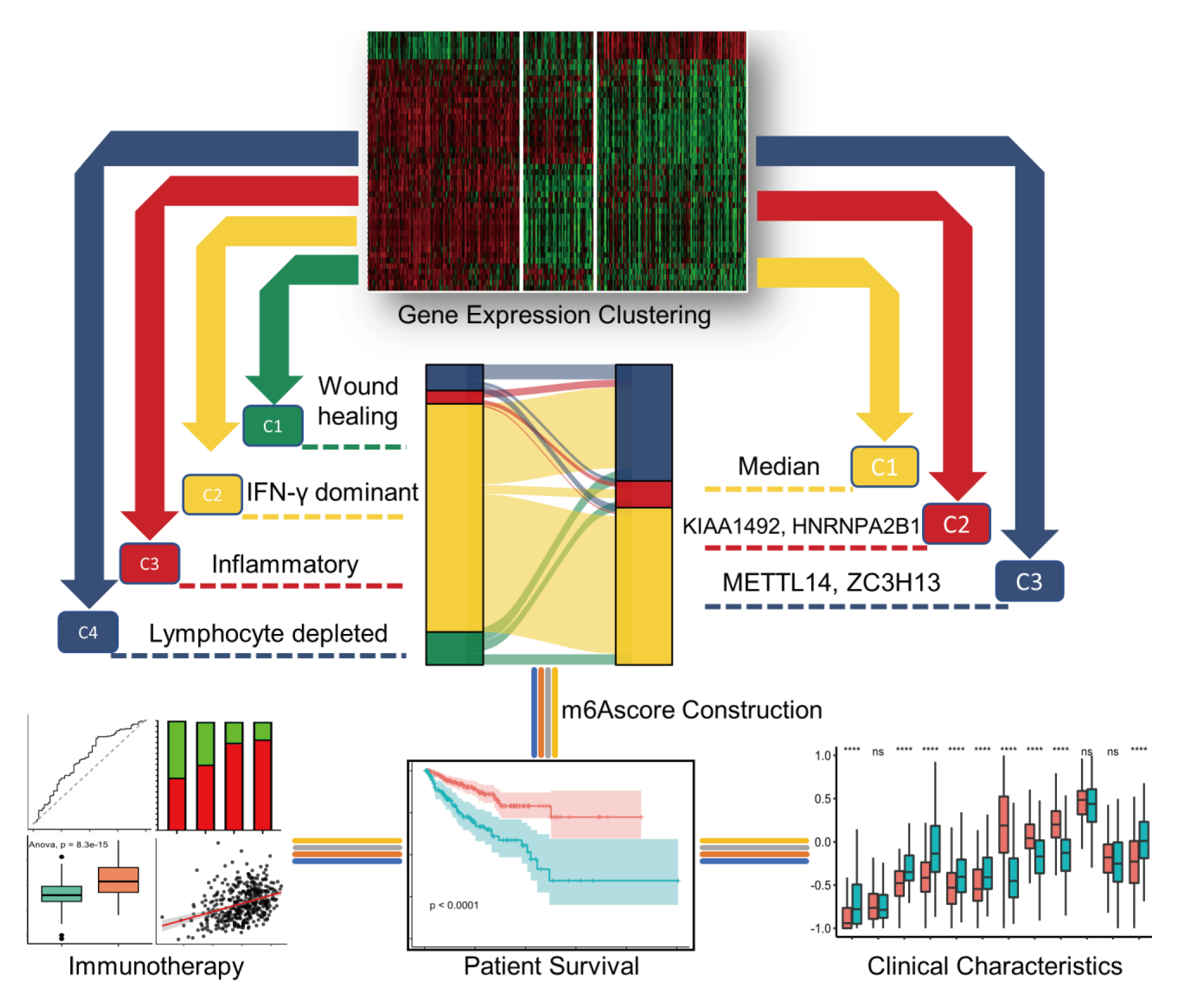

Supplement: Supplementary Figure 1 — Flowchart depicting the workflow of this study. [file Image_1.tif]

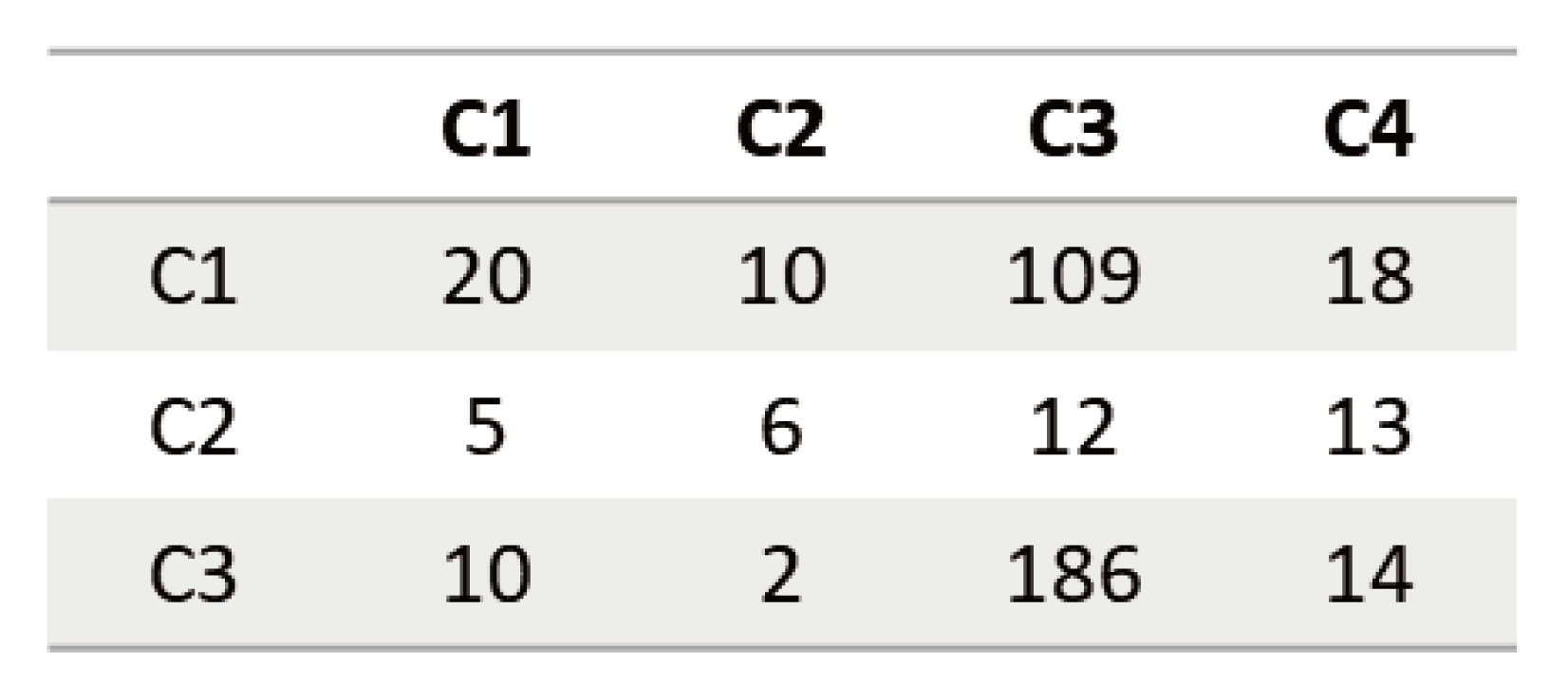

Supplement: Supplementary Figure 2 — Table shown that relationship between m6A regulators clusters and immune clusters in the prostate cancer. colnames are immune clusters. rownames are m6A regulators clusters. [file Image_2.tif]

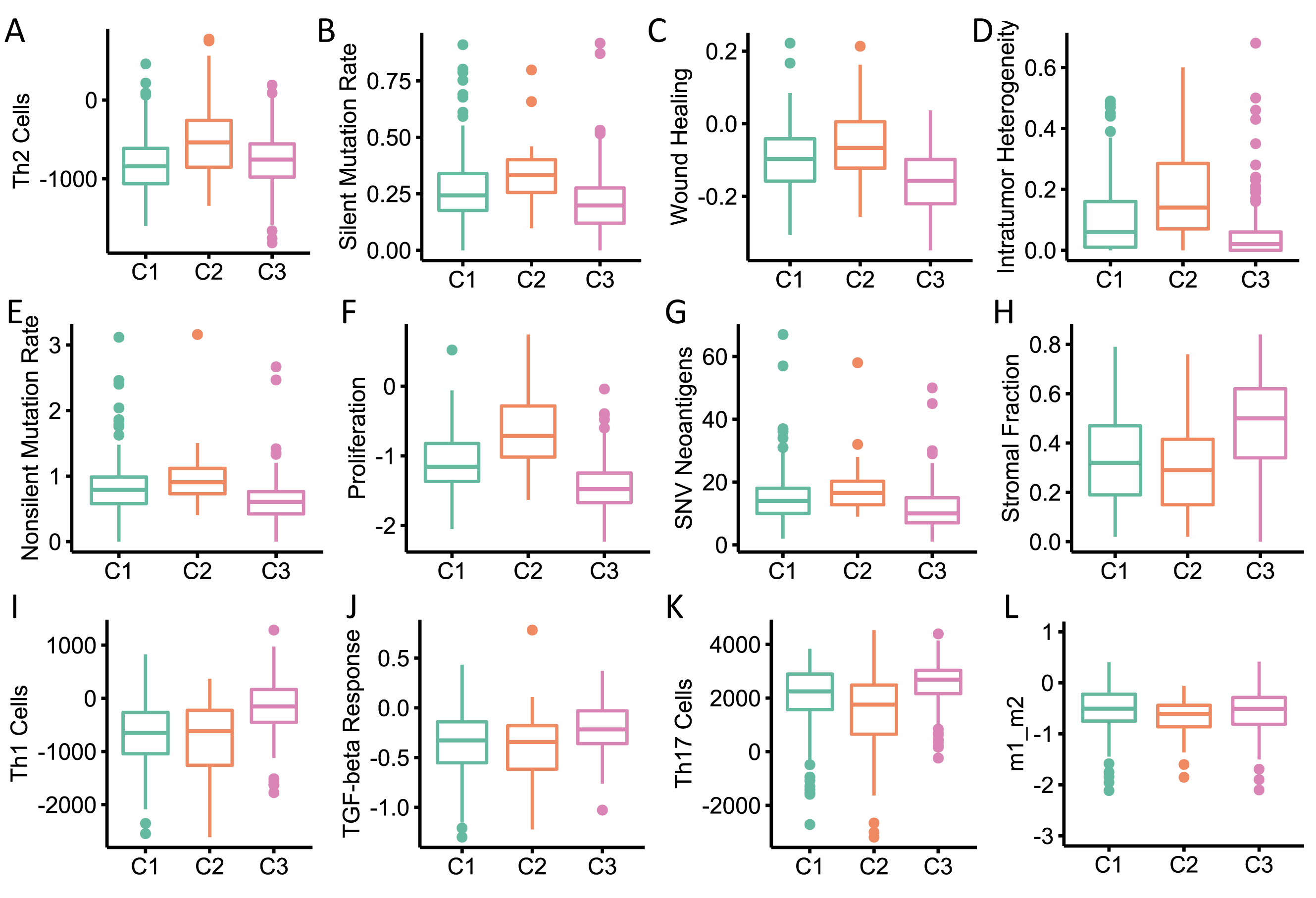

Supplement: Supplementary Figure 3 — The histogram shows the comparison of 12 characteristic scores among three m6A regulators clusters. [file Image_3.tif]

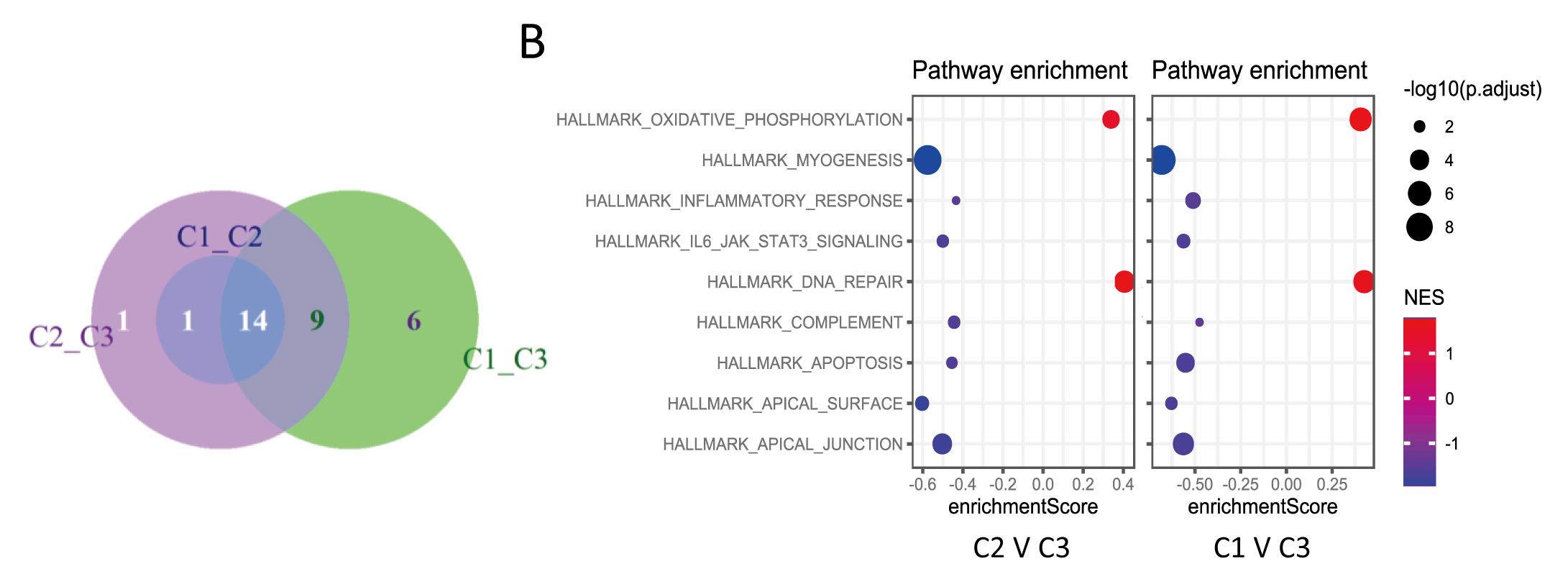

Supplement: Supplementary Figure 4 — (A) Venn Diagram showed that 9 hallmarks were obtained from the intersection. C1_C2 represent the different hallmark between cluster1 and cluster2. C1_C3 represent the different hallmark between cluster1 and cluster3. C2_C3 represent the different hallmark between cluster2 and cluster3. (B) GSEA analysis of 50 hallmarkers. [file Image_4.tif]

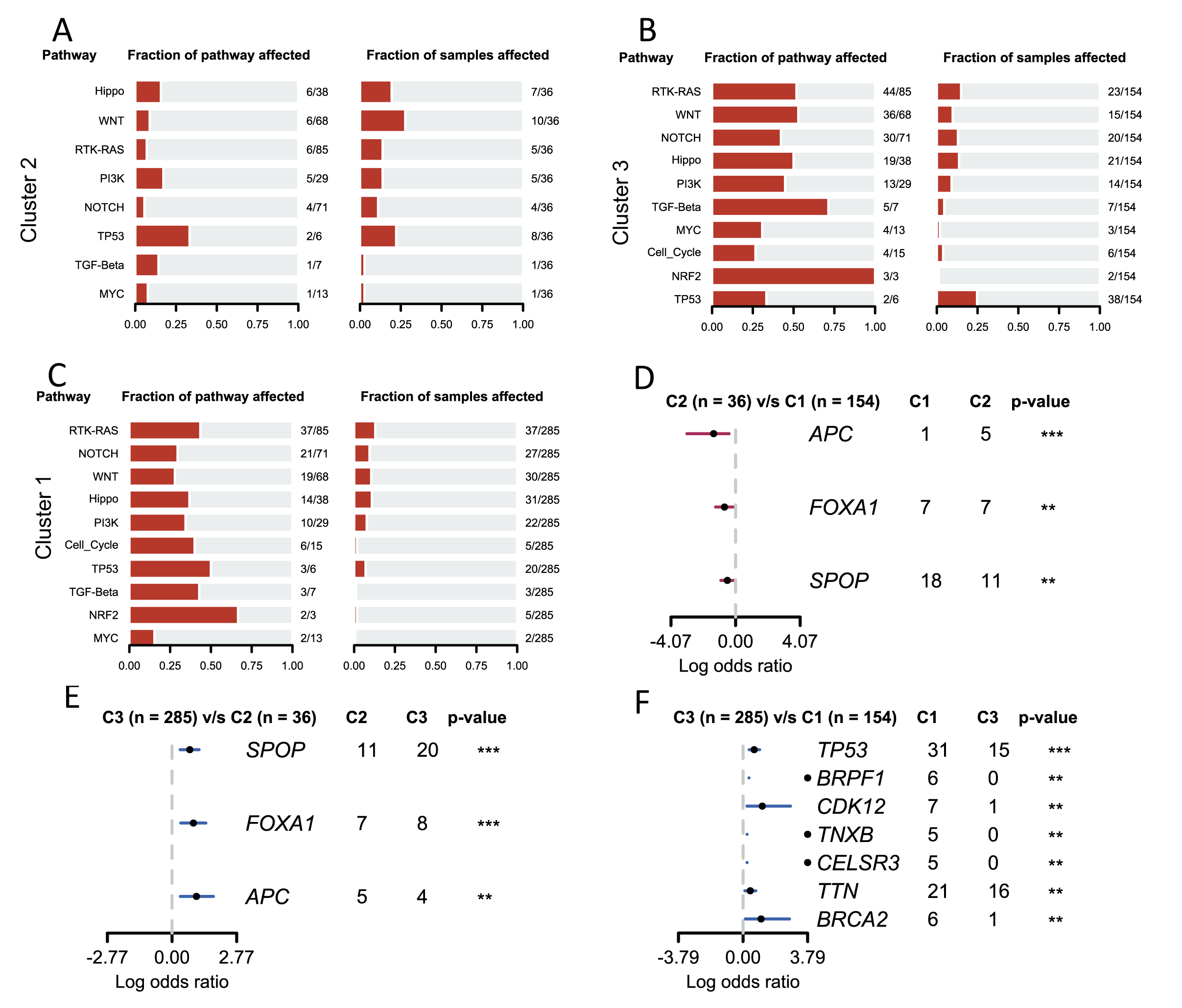

Supplement: Supplementary Figure 5 — (A) Cluster1. The first column represented tumor associate signaling pathway. The second column shown the number and proportion of mutated genes in signaling pathway. The third column indicated mutation patients. (B) Cluster2. (C) Cluster3. (D) The Cluster3 is compared with the Cluster1. Forest plot shown differences of mutation genes among three m6A regulators clusters. median column represented gene mutation frequencies. (** p<0.01; ***p<0.001) (E) The Cluster3 is compared with the Cluster2. (F) The Cluster3 is compared with the Cluster1. [file Image_5.tif]

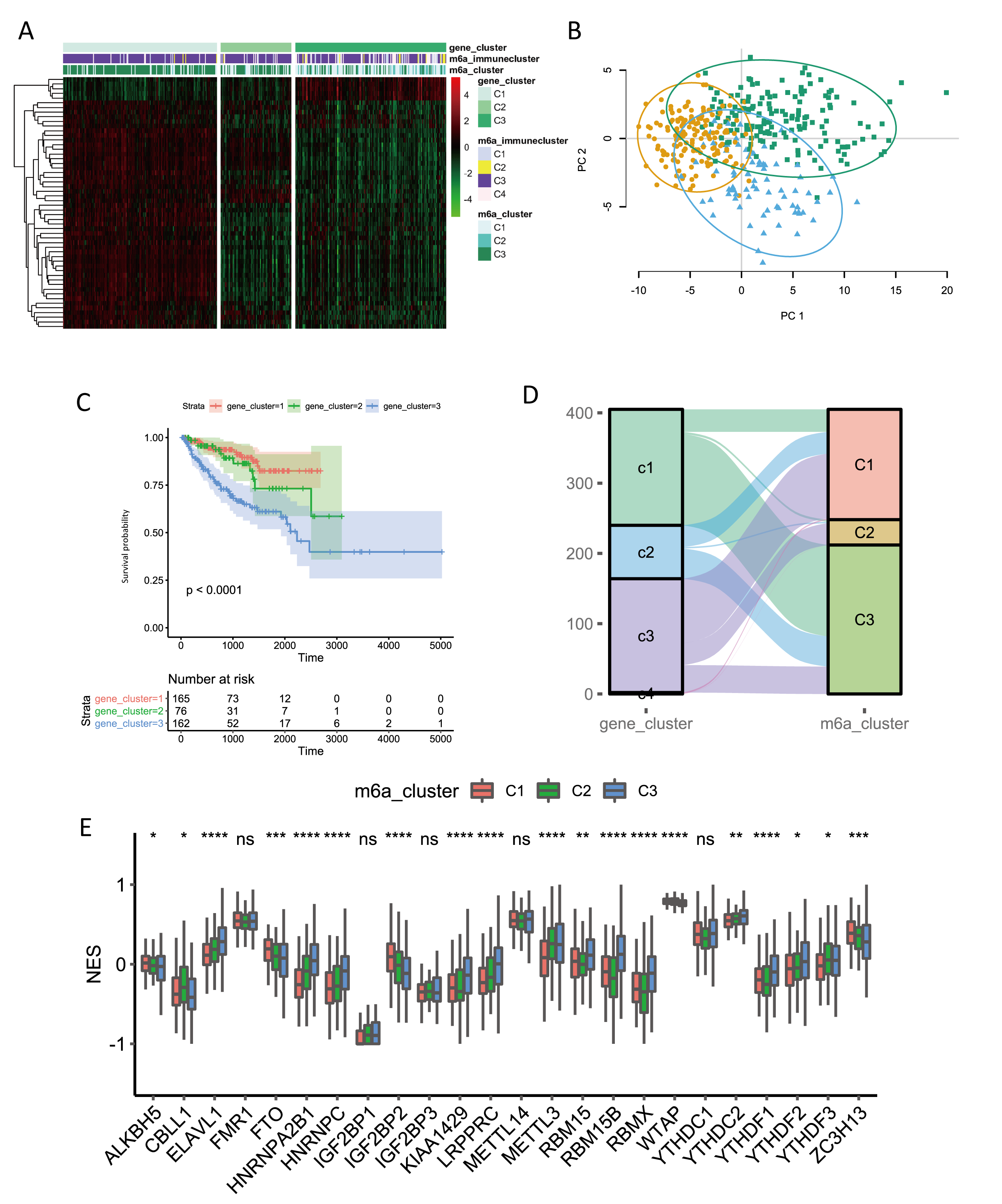

Supplement: Supplementary Figure 6 — Unsupervised clustering of overlapping m6A expression patterns differential genes. (A) Three clusters were identified by unsupervised clustering base on differential genes overlapping of three m6A expression patterns. (B) Principal component analysis shown a remarkable difference among three differential genes clusters. (C) Survival analyses for the three differential genes clusters based on TCGA database including 165 cases in differential genes cluster-1, 76 cases in differential genes cluster-2, 162 cases in differential genes cluster-3. Kaplan-Meier curves with Log-rank p value <0.0001 showed a significant survival difference among three differential genes clusters. The cluster-3 showed significantly badly FPI than the other two cluster. (D) Alluvial diagram showing the changes of m6A regulators clusters and differential genes clusters of the prostate cancer samples. (E) The expression of 24 m6A regulators in three clusters. cluster1, red. cluster2, green, cluster3, blue. (*P<005; **P<001; ***P<0001; ****P<0.0001). [file Image_6.tif]

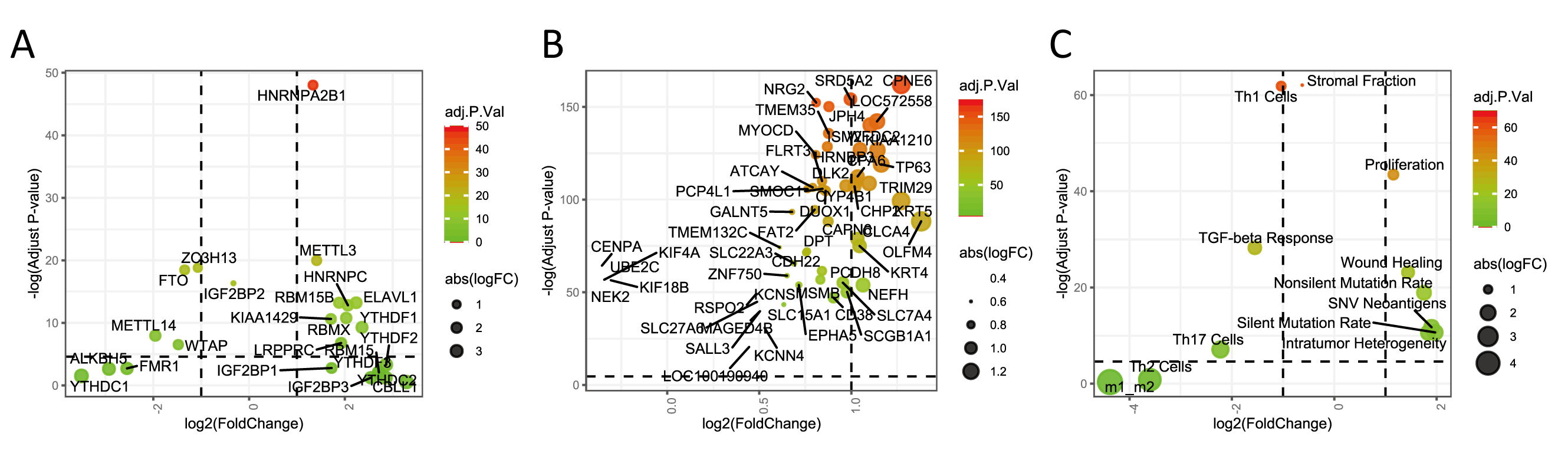

Supplement: Supplementary Figure 7 — (A) bubble shown difference in key characteristics between high and low risk score groups. -log10(adjust p value) were marked with color. log(foldchange) were marked with size. (B) bubble shown difference in expression of 24 m6A genes between high and low risk score groups. (C) bubble shown difference in expression of m6A associated genes between high and low risk score groups. [file Image_7.tif]
